# Biomass-Derived Sustainable Electrode Material for Low-Grade Heat Harvesting

**DOI:** 10.3390/nano13091488

**Published:** 2023-04-27

**Authors:** Jonghak Park, Taewoo Kim

**Affiliations:** Department of Mechanical Engineering, Incheon National University, Incheon 22012, Republic of Korea

**Keywords:** thermocell, thermogalvanic cell, thermo-electrochemical cell, low-grade heat, biomass

## Abstract

The ever-increasing energy demand and global warming caused by fossil fuels push for the exploration of sustainable and eco-friendly energy sources. Waste thermal energy has been considered as one of the promising candidates for sustainable power generation as it is abundantly available everywhere in our daily lives. Recently, thermo-electrochemical cells based on the temperature-dependent redox potential have been intensely studied for efficiently harnessing low-grade waste heat. Despite considerable progress in improving thermocell performance, no attempt was made to develop electrode materials from renewable precursors. In this work, we report the synthesis of a porous carbon electrode from mandarin peel waste through carbonization and activation processes. The influence of carbonization temperature and activating agent/carbon precursor ratio on the performance of thermocell was studied to optimize the microstructure and elemental composition of electrode materials. Due to its well-developed pore structure and nitrogen doping, the mandarin peel-derived electrodes carbonized at 800 °C delivered the maximum power density. The areal power density (*P*) of 193.4 mW m^−2^ and *P/*(Δ*T*)^2^ of 0.236 mW m^−2^ K^−2^ were achieved at Δ*T* of 28.6 K. However, KOH-activated electrodes showed no performance enhancement regardless of activating agent/carbon precursor ratio. The electrode material developed here worked well under different temperature differences, proving its feasibility in harvesting electrical energy from various types of waste heat sources.

## 1. Introduction

Our society needs substantial quantities of energy for manufacturing, transportation, and living. However, unfortunately, most energy has been based on fossil fuels that are not replenished. Furthermore, the release of greenhouse gases from burning fossil fuels is responsible for rising global warming and climate change [1]. To address the environmental issue of fossil fuels and meet growing energy demand, renewable energies, such as solar radiation, wind energy, and geothermal energy, have attracted lots of interest for sustainable power production [2]. Among renewable energies, waste heat energy has gained significant attention by virtue of its abundance and low dependence on weather conditions, assuring continuous and predictable power generation.

**Figure 1:**
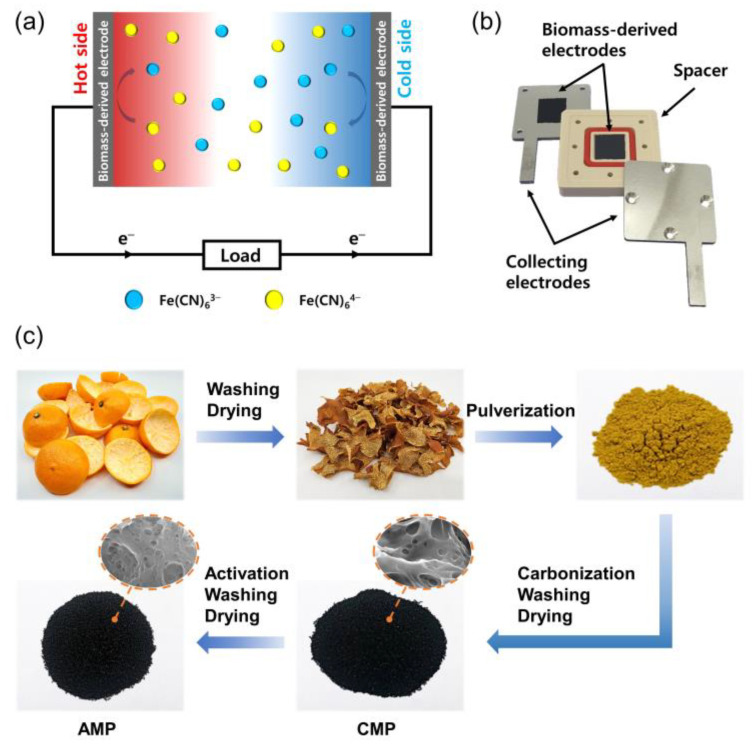
(**a**) Schematic illustration of thermocell operation mechanism, (**b**) optical images of thermocell components, and (**c**) schematic illustration of the synthesis of CMP and AMP from mandarin peel waste.

Recently, thermocells (also called thermo-electrochemical cells), featuring low-cost, simple components, and direct heat-to-electricity conversion, have been considered as one of the promising technologies for waste heat harvesting [3,4,5,6,7]. A typical thermocell, which consists of two identical electrodes and an electrolyte, generates a voltage when the electrodes are at different temperatures, as shown in Figure 1a,b. A redox couple, such as Fe(CN)_6_^3−^/Fe(CN)_6_^4−^ [8], Fe^2+^/Fe^3+^ [9], or I^−^/I_3_^−^ [10], is involved in the reactions on both electrodes, implying that the standard redox potentials for them are the same. The open-circuit voltage (*V*_oc_) of a thermocell relies on the temperature coefficient of redox potential (*α*), also known as ionic Seebeck coefficient, which can be written as [11]:

(1) $$\alpha =\frac{\partial E}{\partial T}=\frac{\Delta S_{\mathrm{rxn}}}{nF}$$

(2) $$V_{\mathrm{OC}}=\alpha \Delta T$$

where *E* is the redox potential of a given redox couple, Δ*S*_rxn_ is the entropy change in the reaction, *n* is the number of electrons transferred, *F* is Faraday’s constant, *T* is the temperature at which the reaction occurs, and Δ*T* denotes the temperature difference between the two electrodes. In particular, thermocells have outstanding *α* values (−1.4 mV K^−1^ and 1.76 mV K^−1^ for Fe(CN)_6_^3−^/Fe(CN)_6_^4−^ [12,13] and Fe^2+^/Fe^3+^ [14], respectively) much higher than the Seebeck coefficient of conventional thermoelectrics (≈100 μV K^−1^) [15], which makes them attractive for harnessing low-grade heat (<150 °C) that amounts to over 60% of waste heat [16].

Given the wide distribution and small temperature difference with the environment of low-grade heat, it is essential to make a large-scale device that requires inexpensive electrodes from abundant and cheap sources. Efforts have been dedicated to preparing inexpensive and high-performance electrode materials such as graphene [17,18], carbon nanotube [12,17,18,19,20,21], and carbon cloth [22]. More recently, Kim et al. reported thermocell operation with K_3_Fe(CN)_6_/K_4_Fe(CN)_6_ electrolyte under alkaline conditions, which enables the use of non-noble metals such as copper and nickel that have not been used due to the corrosion with the electrolyte of thermocells [23,24]. Considering that metal production for renewable energy can be a new threat to the environment [25], it is urgent to explore sustainable sources for electrode materials. Given that a thermocell electrode requires corrosion resistance, good charge transfer characteristics, and a hierarchical porous structure, 3D porous carbonaceous material would be a promising candidate for thermocell electrodes. Biomass-derived carbon materials, readily available and abundant, are expected to allow the fabrication of large-scale thermocell for practical applications. In recent years, porous carbon materials derived from biomass waste have gained considerable attention as capacitors [26,27,28,29,30,31,32,33], batteries [34,35,36,37], and adsorbents [38,39] owing to low cost, eco-friendliness, and sustainability. Agricultural biomasses such as peanut shell [32], walnut shell [31], orange peel [27,33], jujube [28], tea waste [29], coffee waste [40], bagasse [30,34], leaf [38], and cone [26] have been proposed as sustainable sources for synthesizing hierarchical porous carbons. To our knowledge, however, biomass-derived electrodes for thermocells have not yet been reported.

The research presented in this paper aims to develop a biomass-derived electrode for cost-effective and high-performance thermocells. We proposed a facile and environmentally friendly approach for synthesizing a porous carbon electrode prepared by carbonization and activation treatments of mandarin peel waste. A large number of mandarin peel waste generated from juice production are discarded, and their high biological oxygen demand and chemical oxygen demand lead to soil degradation and environmental pollution [41]. Therefore, using mandarin peel as a carbon precursor would contribute to environmental protection and provide suitable feedstock for electrode materials. The effect of carbonization temperature on the performance of biomass-derived carbon electrodes was investigated and discussed based on their structure and heteroatom doping. Furthermore, we studied the influence of the activating agent/carbon precursor ratio on the electrochemical properties of the electrodes.

## 2. Materials and Methods

### 2.1. Preparation of CMP and AMP from Mandarin Peel Waste

This work used Satsuma mandarin peel waste as a carbon precursor to prepare electrode materials through carbonization and activation processes. First, the mandarin peels were washed several times with deionized (DI) water to remove impurities, followed by drying in a forced air oven at 80 °C for 12 h. Afterward, the dried peels were ground and sieved to obtain mandarin peel powder with a particle size between 75 μm and 120 μm, named DMP. Next, the DMP was carbonized at 600, 700, 800, 900 and 1000 °C at low pressure with a nitrogen (N_2_) flow of 100 sccm for 2 h in a quartz tube furnace at a heating rate of 5 °C min^−1^. Subsequently, the carbonized DMPs were immersed in 0.1 M hydrochloric acid (HCl) solution (Daejung, Siheung, Republic of Korea), stirred for 30 min, and washed with DI water several times to neutralize them and remove any impurities. Finally, the products were dried in a vacuum oven at 80 °C for 12 h. Based on their carbonization temperature, the obtained products were denoted as CMP-600, CMP-700, CMP-800, CMP-900 and CMP-1000.

The mixtures with different weight ratios of KOH and CMP-800 (1:1, 2:1 and 3:1) were prepared by mixing and grinding CMP and KOH flakes (Daejung) using an agate mortar and pestle to ensure homogeneity. The obtained mixtures were heated at 800 °C at low pressure with a N_2_ flow of 100 sccm for 2 h in the tube furnace to activate CMPs. Afterward, the products were washed and dried in the same way as CMP. The activated CMPs with the ratios of 1:1, 2:1, and 3:1 (KOH:CMP-800, *w*/*w*) were denoted as AMP-800-1, AMP-800-2, and AMP-800-3, respectively.

### 2.2. Material Characterization

The surface morphology and microstructure of CMP and AMP samples were observed using field emission scanning microscopes (FESEM, JSM-7001F and 7800F, JEOL, Tokyo, Japan). The surface elemental composition of the samples was determined by energy dispersive X-ray spectroscopy (EDS) with an analyzer (Xmax-50, Oxford Instruments, Abingdon, UK) attached to the FESEM. X-ray photoelectron spectroscopy (XPS) was performed to investigate the surface functional groups and the chemical composition of CMP and AMP using a PHI 5000 Versa Probe Ⅱ (Ulvac, Chigasaki, Japan). X-ray diffraction (XRD) spectra were measured in the 2*θ* range of 10–80° with an X-ray diffractometer (SmartLab, Rigaku, Tokyo, Japan) using Cu Kβ radiation (*λ* = 1.3922 Å) to identify the crystalline structures of CMP and AMP. Raman spectroscopy was performed at an excitation wavelength of 532 nm using a micro-Raman spectrometer (alpha300, Witec, Abingdon, UK). The specific surface areas (SSA) of CMP and AMP samples were measured using the methylene blue adsorption method [42]. Approximately 10 mg of a sample was dispersed in 15 mL of 0.2 mM of methylene blue (Sigma-Aldrich, St. Louis, MO, USA) solution, followed by stirring for 12 h. The obtained solution was centrifuged, and the methylene blue concentration of the supernatant was measured using a UV-Vis spectrometer (Lambda 750, Perkin Elmer, Waltham, MA, USA) to estimate the amount of unadsorbed methylene blue molecules. The SSA of the sample was determined considering the amount of adsorbed methylene blue molecules and the mass of the sample.

### 2.3. Fabrication of Thermocells

CMP (or AMP), Super P, and polyvinylidene fluoride (Sigma-Aldrich) were mixed in N-Methyl-2-pyrrolidone (Daejung) with a weight ratio of 80:15:5 using a ball miller (Pulverisette 23, Fritsch, Idar-Oberstein, Germany) to prepare a homogeneous slurry. It was then coated onto a stainless steel sheet (0.1 mm thickness, Nilaco corporation, Tokyo, Japan) using an adjustable film applicator, followed by a drying process under vacuum at 40 °C for 12 h. The obtained sample was cut into several pieces with a 1 cm × 1 cm dimension for use as electrodes. The potassium hexacyanoferrate (Fujifilm Wako Pure Chemical Corporation, Osaka, Japan) and potassium hexacyanoferrate(II) trihydrate (Daejung) were dissolved in DI water to prepare a K_3_Fe(CN)_6_/K_4_Fe(CN)_6_ electrolyte for thermocell performance tests. A thermocell was assembled with two identical electrodes, stainless steel current collectors, and a 5 mm thick spacer containing an electrolyte (Figure 1b).

### 2.4. Electrochemical Measurements

The *α* of the redox potential of Fe(CN)_6_^3−^/Fe(CN)_6_^4−^ was determined using a U-shaped cell comprising two half-cells to prevent heat conduction between them (Figure S1). The temperatures of the half cells were controlled by circulating hot and cold water through the jackets surrounding the cells. The voltage and the temperature difference between the half-cells were monitored with a digital multimeter (2000, Keithley, Cleveland, OH, USA) and a data logger (GL220, Graphtec Corporation, Tokyo, Japan).

The thermocell was placed between hot and cold blocks to make a temperature difference between the electrodes. These blocks were connected to refrigerated/heated bath circulators (RW3-1025P, Jeiotech, Daejeon, Republic of Korea), and their temperatures were controlled with an accuracy of ±0.05 °C. The output electrical power of the thermocell was measured by the digital multimeter with different load resistances. The load resistances were controlled using a scanner card (2000-SCAN, Keithley, Cleveland, OH, USA) mounted in the multimeter considering the internal resistance of the thermocell. The cyclic voltammetry and electrochemical impedance spectroscopy (EIS) were conducted using a potentiostat/galvanostat (Autolab PGSTAT302N, Metrohm, Herisau, Switzerland) to examine the electrochemical properties of the electrodes. The cyclic voltammetry was performed with a three-electrode setup consisting of a CMP (or AMP) working electrode, a platinum counter electrode, and an Ag/AgCl reference electrode with 3.5 M KCl. The electrolyte was 5 mM of K_3_Fe(CN)_6_ and 0.1 M of KCl solution.

The ionic conductivity of the electrolyte was measured at different temperatures (Figure S2) using a conductivity meter (S230, Mettler Toledo, Greifensee, Switzerland). It increased linearly from 180 to 243 mS cm^−1^ as the temperature increased from 20 to 40 °C. The thermal conductivity of the electrolyte was ~0.55 W m^−1^ K^−1^ [43].

## 3. Results

Figure 1c shows the schematic diagram for synthesizing CMP and AMP from mandarin peel waste. Firstly, the peels were dried and pulverized to obtain DMP. After that, it was carbonized in an inert atmosphere, converting typical components of the peel, pectin, cellulose, hemicellulose, and lignin into carbon materials (CMP). AMP was made from CMP-800 by chemical activation with KOH, which provided a hierarchical 3D porous carbon structure. The pore formation mechanism of KOH activation is generally considered as the chemical reaction between KOH and carbon (6KOH + 2C → 2K + 3H_2_ + 2K_2_CO_3_). K_2_CO_3_ decomposes into K_2_O and CO_2_ at a temperature above 700 °C. K_2_CO_3_/K_2_O/CO_2_ further reacts with carbon atoms, introducing pores into carbon materials [32,33].

**Figure 2:**
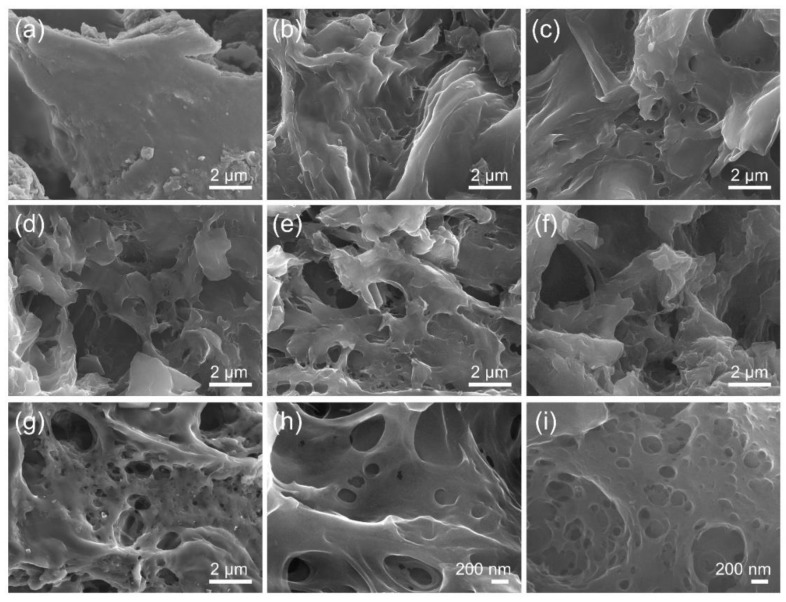
FESEM images of (**a**) DMP, (**b**) CMP-600, (**c**) CMP-700, (**d**) CMP-800, (**e**) CMP-900, (**f**) CMP-1000, (**g**) AMP-800-3, and images of (**h**) CMP-800 and (**i**) AMP-800-3 at a different resolution.

The morphology and microstructure of DMP, CMP, and AMP were examined by SEM analysis, as presented in Figure 2. It is found in Figure 2a that DMP possessed a smooth surface without noticeable pores and cracks. As the carbonization temperature increased from 600 °C to 1000 °C, a pore structure was developed as shown in Figure 2b–f, which is ascribed to the elimination of heteroatoms as volatile gases [44] and the removal of salts such as K_2_O, MgO, and CaO in the washing process. Salt particles of various sizes were observed in as-synthesized CMP that was not washed after the carbonization (Figure S3, the composition of the particles and concentrations will be discussed later). These salts were mostly removed after washing with HCl and DI water, leaving a hierarchical porous structure. CMP-800, 900, and 1000 had a 3D hierarchical structure with rough and porous surfaces, which can facilitate ion transfer and enlarge the accessible surface area, thereby enhancing the electrochemical performance of electrode materials [30,33,34]. The effect of KOH chemical activation on the structure of CMP can be observed by comparing the images of CMP-800 and AMP-800 samples. AMP-800-3 showed a similar porous structure as CMP-800, but there were tens of nanometer-sized pores on its surface, which was not observed in CMP-800, as shown in Figure 2h,i. It can be confirmed from the SEM analysis that a 3D porous structure was made by carbonization, and the chemical activation process introduced tiny pores.

**Figure 3:**
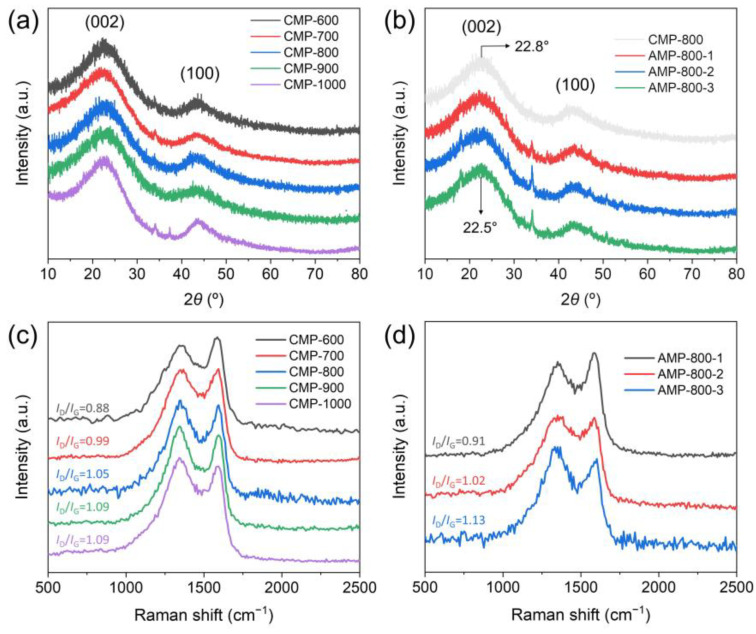
XRD patterns of (**a**) CMP and (**b**) AMP, and Raman spectra of (**c**) CMP and (**d**) AMP.

The crystal structures of the prepared samples were analyzed by XRD, and the results are shown in Figure 3a,b. All the XRD patterns showed broad diffraction peaks at around 22.8° and 43.5° belonging to the (002) and (100) planes of graphitic carbon [31]. According to the Bragg equation, the interlayer distance of CMP samples was calculated to be 0.352 nm, which is higher than that of graphite (0.335 nm), indicating their low graphitization and high defect density. No apparent shift of the (002) peak position was observed depending on the carbonization temperatures, revealing that the interplanar spacing was not affected by the carbonization temperature in the range of 600–1000 °C. CMP-1000 exhibited a relatively low full-width half maximum (FWHM), ascribed to its relatively high crystallinity due to the higher processing temperature. AMP-800 samples had (002) peak slightly shifted to 22.5°, suggesting their slightly larger interlayer spacing (0.357 nm) compared to CMP samples (Figure 3b). The expansion of the interplanar spacing between carbon layers by KOH activation is consistent with the previous literature [38,45]. The microstructure of CMP and AMP was further analyzed by Raman spectroscopy. Figure 3c shows the Raman spectra of CMP samples. All spectra exhibited two typical distinct peaks at ~1350 cm^−1^ and ~1590 cm^−1^, known as D and G peaks. The D peak is associated with the defects-induced breathing mode of sp^2^ rings, while the G peak corresponds to the in-plane vibrational mode of sp^2^ carbon atoms [46]. The intensity ratio of the D to G peak (*I*_D_/*I*_G_) demonstrates the degree of defect and disorder of carbon materials. The ratio *I*_D_/*I*_G_ of CMP increased from 0.88 to 1.09 as the carbonization temperature changed from 600 to 1000 °C. During the carbonization process, heteroatoms such as oxygen and nitrogen abundant in biomass feedstocks are eliminated, and the residual carbon atoms form a turbostratic structure consisting of nanometer-sized polyaromatic layers [47,48]. The boundaries of the polyaromatic layers contribute to the increase in the D peak intensity. This tendency agrees with the XPS results discussed below, which prove that the oxygen and nitrogen content decreased with the increase in carbonization temperature. The *I*_D_/*I*_G_ of Raman spectra of AMP, as presented in Figure 3d, increased as the ratio of KOH:CMP increased, implying that the chemical activation induced the defects and disorders of structures in the samples [31,49,50].

**Figure 4:**
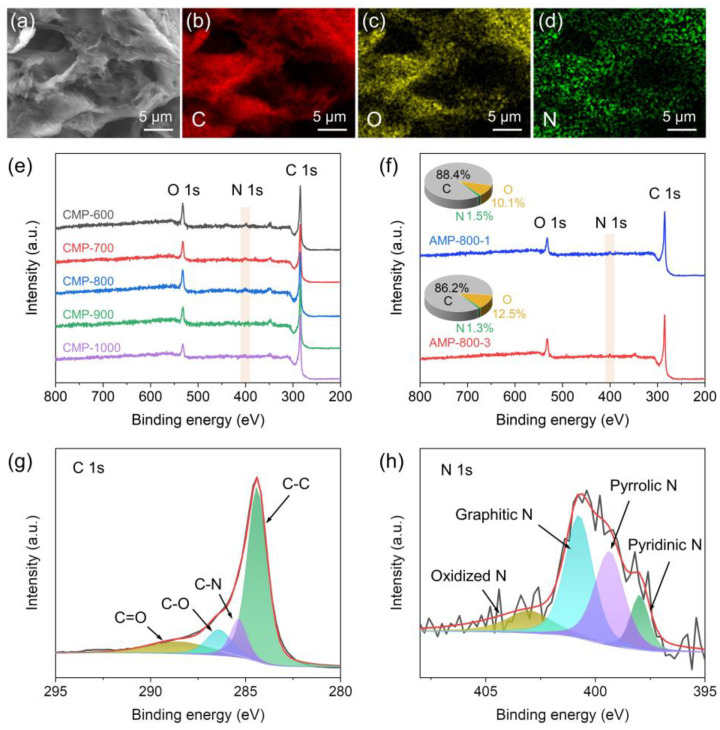
(**a**) FESEM and (**b**–**d**) EDS mapping images of CMP-900, XPS survey spectra of (**e**) CMP and (**f**) AMP samples, high-resolution XPS spectra of (**g**) C 1s and (**h**) N 1s of CMP-800.

CMP and AMP were analyzed by EDS in order to determine their elemental composition and distribution. The SEM and EDS mapping images shown in Figure 4a–d reveal the uniform distribution of C, O, and N elements on the surface of CMP, confirming O and N-doping on carbon. AMP also exhibited a homogeneous distribution of the elements throughout its surface (Figure S4). It has been well recognized that nitrogen doping is an efficient way to enhance the electrical conductivity and the wettability of carbon materials [29,51], which play an essential role in improving the performance of electrochemical cells. The surface composition and chemical bonds of CMP samples were characterized by XPS, and the results are displayed in Figure 4e,g,h. The survey spectra confirmed the presence of C, O, and N elements in all the samples, as shown in Figure 4e. The relative atomic concentrations of the elements are summarized in Table 1. In increasing the carbonization temperature, the content of carbon increased, but the oxygen and nitrogen content decreased. The high-resolution C 1s spectrum of CMP-800 could be fitted to four peaks assigned to C-C (284.8 eV), C-N (285.8 eV), C-O (286.8 eV), and C=O (288.9 eV) bonds [26,30] (Figure 4g). As shown in Figure 4h, the high-resolution N 1s spectrum also were deconvoluted into four peaks at 398.4, 399.8, 401.2, and 403.4 eV, which are attributed to pyridinic N, pyrrolic N, graphitic N (or quaternary N), and oxidized N, respectively [26,30,52]. The results revealed that the nitrogen atoms were successfully doped into the porous carbon matrix. The graphitic N (40.6%) and pyrrolic N (32.7%) accounted for most N bonding configurations. The graphitic N plays a vital role in enhancing the electrical conductivity and charge transfer of carbonaceous materials [51,53]. Pyrrolic N, the nitrogen atom substituting a carbon atom in the five-membered ring, provides high charge mobility [54,55,56]. As displayed in their XPS spectra (Figure 4f), AMP samples also contained C, O, and N elements.

XPS was also examined to probe the elemental composition of the as-synthesized CMP-800 sample, as shown in Figure S5. Since the sample was not washed with HCl and DI water, it possessed a large amount of potassium, magnesium, and calcium, which are abundant elements in mandarin peel [57,58]. The surface atomic concentrations of C, O, K, Mg and Ca in the as-synthesized CMP-800 were determined to be 27.6, 50.5, 6.4, 11.4 and 4.1%, respectively, confirming that the heteroatoms covered most of the surface of the CMP. Considering the XPS analysis results, the particles observed on the surface of the as-synthesized CMP might be composed of K, Mg, Ca and O elements (Figure S3). After washing, the concentration of C increased to 86.3%, and that of O decreased to 12.1% (Figure S5), and most of K, Mg, and Ca disappeared, which agrees well with the SEM result in Figure 2d.

**Figure 5:**
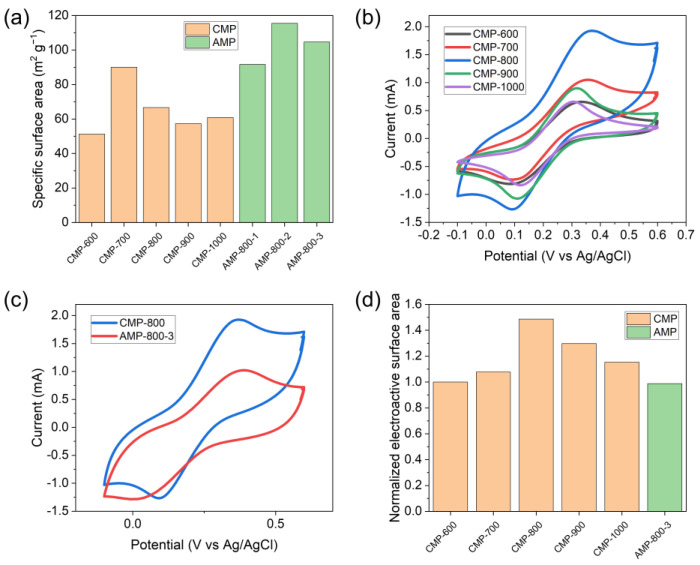
(**a**) The specific surface area of CMP and AMP, (**b**) cyclic voltammograms of CMP, (**c**) cyclic voltammograms of CMP-800 and AMP-800-3, and (**d**) the electroactive surface area of CMP and AMP normalized by that of CMP-600.

Figure 5a shows the SSA of CMP and AMP, measured by the methylene blue adsorption method. The SSA of CMP was increased from 51.2 to 90.0 m^2^ g^−1^ as the carbonization temperature raised to 700 °C and decreased to 60.0 m^2^ g^−1^ with the further temperature increase due to the pore generation and widening. Cyclic voltammograms (Figure 5b) show that the carbonization temperature affected the peak current. According to the Randles–Sevcik equation, the electroactive surface area (ESA) is expressed by [59]:

(3) $$I_{p}=268600AD^{1/2}n^{3/2}v^{1/2}C$$

where *I*_p_ is the faradaic peak current, *A* is the ESA, *D* is the diffusion coefficient of Fe(CN)_6_^3−^, *n* is the number of electrons transferred in the redox reaction, *v* is the potential scan rate, and *C* is the concentration of the reaction species (Fe(CN)_6_^3−^) in the electrolyte. Therefore, the ESA is proportional to the peak current. The ESA of CMP normalized by that of CMP-600 is plotted in Figure 5d, where CMP-800 showed the maximum value. Although CMP-700 had the highest SSA, the large pores generated in CMP-800 (Figure 2d) may play a role in enhancing the ESA by facilitating ion transport.

The cyclic voltammogram of AMP-800-3 showed a smaller peak current than CMP-800 despite its higher SSA (Figure 5c). Therefore, its ESA was also estimated to be smaller than that of CMP-800, as shown in Figure 5d. It can be ascribed to an irreversible charge transfer at the AMP-800-3 electrode, related to its high peak-to-peak separation in the voltammogram.

**Figure 6:**
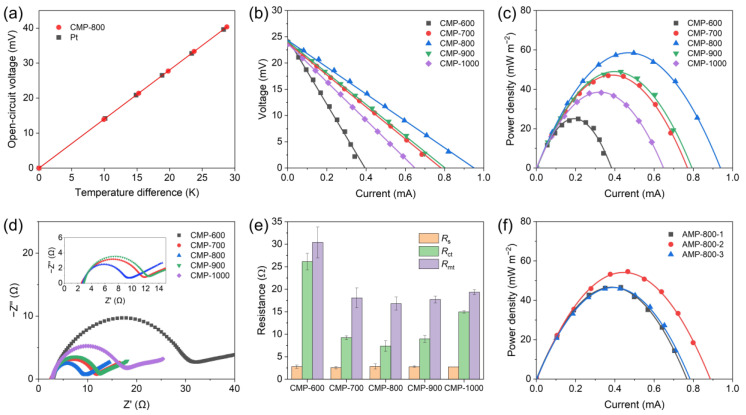
(**a**) The dependence of open-circuit voltage of the thermocells on the temperature difference between the hot and cold electrodes, (**b**) voltage–current curves, (**c**) areal power density–current curves, (**d**) Nyquist plots, (**e**) internal resistances of CMP electrodes, and (**f**) areal power density–current curves of AMP electrodes.

The *α* of Fe(CN)_6_^3−^/Fe(CN)_6_^4−^ redox couple was measured using a U-shaped thermocell comprising CMP-800 electrodes and 0.4 M K_3_Fe(CN)_6_/K_4_Fe(CN)_6_ electrolyte (Figure S1). For comparison, the *α* was also determined using a thermocell composed of platinum electrodes. Figure 6a shows the *V*_oc_ of the thermocells with respect to the Δ*T* between the hot and cold electrodes, exhibiting a linear relationship between them over a range of temperature differences from 0 °C to 28.6 °C. The slope of the fitted line gives the estimate of *α* = −1.40 mV K^−1^ for both the CMP and Pt electrodes, which is consistent with the value reported in the literature for Fe(CN)_6_^3−^/Fe(CN)_6_^4−^ redox couple [12,13]. The minus sign of *α* indicates that the cold electrode has a higher potential than the hot electrode, and Δ*S*_rxn_ of the redox couple is negative.

The power generation performance of CMP and AMP electrodes was investigated by measuring the voltage between the hot and cold electrodes under different external resistances. The temperatures of the hot and cold blocks were controlled at 45 °C and 15 °C. As plotted in Figure 6b, the *V*_oc_ of CMP electrodes was 24.4 ± 0.18 mV, indicating that the Δ*T* between the electrodes was about 17.4 °C which was calculated from the *V*_oc_ and *α* measured above. A temperature drop can arise from the thermal interface resistances at hot (cold) block/collecting electrode/CMP electrode interfaces and their thermal resistances, which reduces the Δ*T* between the electrodes.

CMP-800 showed the lowest internal resistance of 27.0 Ω obtained from the voltage–current curve slope. The corresponding areal power density–current curve plotted in Figure 6c confirmed that CMP-800 showed the maximum power density owing to its lowest internal resistance. CMP-700 and 900 had slightly lower performance, and CMP-600 and 1000 exhibited poor performance along with higher internal resistance. The reason lies in the synergistic effect of pore structure and heteroatoms doping of CMP, where the former facilitates ion transport and enlarges active surface area, and the latter improves the electrical conductivity and charge transfer characteristic of carbonaceous materials. The porosity of CMP increased with the carbonization temperature increasing from 600 °C to 800 °C, while no visible difference was observed among CMP-800, 900, and 1000 samples (Figure 2b–f). It has been reported that the amount of pores and the surface area of biomass increase until the carbonization temperature reaches 900 °C and decreases above 900 °C due to pore widening and the coalescence of neighboring pores [39]. The nitrogen/carbon ratio of CMP remained at a constant value of ~2% until 800 °C and dramatically decreased to below 1% above 900 °C (Table 1). CMP-600 and 700 possessed relatively poor pore structure, whereas CMP-900 and 1000 had lower nitrogen content than CMP-800. Thus, CMP-800 owed its higher performance to the synergistic effect of well-developed pore structure and nitrogen doping.

The cell was repeatedly assembled and disassembled to prove the robustness of CMP electrodes. No delamination and crack were observed after ten assemblies and performance tests, as shown in Figure S6a,b. The power density and internal resistance of thermocell with CMP electrodes maintained their values without a noticeable change, confirming the robustness of CMP electrodes.

The internal resistance of thermocell consists of three resistances: ohmic resistance (*R*_s_), charge transfer resistance (*R*_ct_), and mass transport resistance (*R*_mt_) [60]. EIS was employed to investigate these three resistances of CMP electrodes further. The *R*_s_ and *R*_ct_ were calculated from EIS results using an equivalent circuit model (Figure S7). Nyquist plots depicted in Figure 6d confirmed that all the CMP samples had a similar *R*_s_ of 2.7 ± 0.1 Ω. However, the carbonization temperature significantly influenced the *R*_ct_, corresponding to the diameter of the semicircle. Three internal resistances are summarized in Figure 6e, where the *R*_mt_ was obtained by subtracting *R*_s_ and *R*_ct_ from the total internal resistance of the thermocell. It can be seen that CMP-800 possessed the lowest *R*_ct_ and *R*_mt_ owing to its high porosity and nitrogen content. CMP-1000 had slightly higher *R*_mt_ than CMP-700, 800, and 900 but much higher *R*_ct_, which implies that the low performance originated from poor charge transfer property due to its low nitrogen content. The areal power densities of AMP-800-1, 2, and 3 were similar to or lower than that of CMP-800 (Figure 6f). Although the KOH activation enhanced the SSA of AMP, it deteriorated the charge transfer characteristics of AMP by destroying the graphitic structure and decreasing the conductivity of AMP [61,62], as confirmed in its cyclic voltammogram (Figure 5c). Given the results in Figure 5a,c and Figure 6c,f, the improvement of SSA and the degradation of charge transfer characteristics produced a thermocell performance similar to that of CMP.

**Figure 7:**
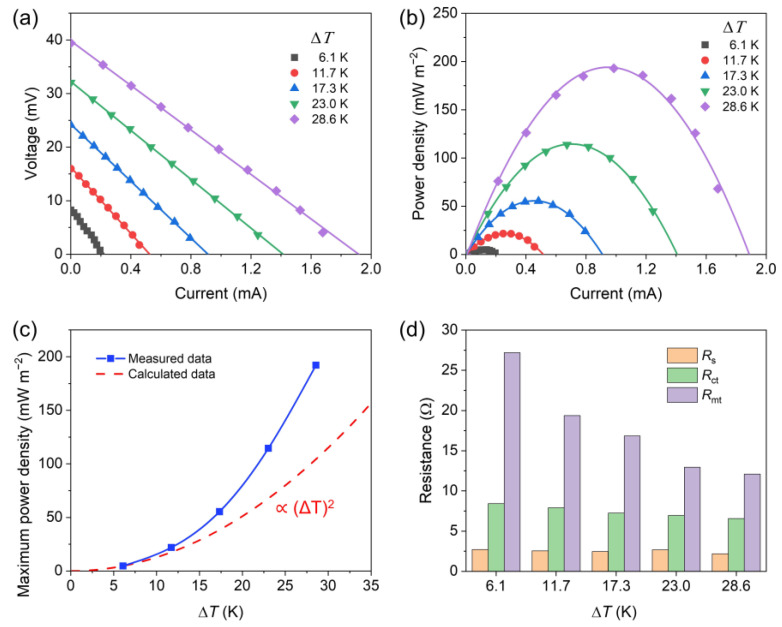
(**a**) Voltage–current curves, (**b**) corresponding areal power density–current curves, (**c**) maximum power density–Δ*T* curves, and (**d**) internal resistances of CMP-800 electrodes as a function of Δ*T*.

Figure 7a,b provide the voltage–current curves and corresponding power–current curves of CMP-800 under different temperature differences. The temperature of the cold block was fixed at 15 °C, and that of the hot block was elevated to change Δ*T*. The open-circuit voltage increased with increasing Δ*T*, and the slope of the voltage–current line declined, revealing that the internal resistance decreased as Δ*T* increased. The maximum power of thermocell can be expressed as

(4) $$P_{\max}=\frac{V_{\mathrm{OC}}{}^{2}}{4R_{\mathrm{int}}}=\frac{{\left(\alpha \Delta T\right)}^{2}}{4R_{\mathrm{int}}}$$

where *R*_int_ denotes the internal resistance of thermocell. It can be found from Figure 7c that the maximum power density of CMP deviates from the calculated curve that is proportional to (Δ*T*)^2^, which is attributed to the decrease in *R*_int_. The three primary internal resistances are displayed in Figure 7d to further explore the change of *R*_int_. The resistances were estimated from the voltage–current curves in Figure 7a and the EIS results shown in Figure S8. The decrease in the internal resistance was mainly due to the decrease in *R*_mt_ that can be reduced by the temperature gradient-induced convection flow of electrolyte. Figure 7 proves that CMP electrodes can efficiently harvest electrical energy from various types of low-grade heat sources with different temperatures.

The areal power density (*P*) was 193.4 mW m^−2^ and *P/*(Δ*T*)^2^ was 0.236 mW m^−2^ K^−2^ for Δ*T* of 28.6 K (Figure 7b), which are comparable to those of MXene/SWCNTs/PANI composite (0.0822 mW m^−2^ K^−2^ at Δ*T* of 40 K) [63], CNT (0.353 mW m^−2^ K^−2^ at Δ*T* of 50 K) [64], CNT/graphene electrode (0.460 mW m^−2^ K^−2^ at Δ*T* of 50 K) [64], N-doped CNT/Pd composite (0.432 mW m^−2^ K^−2^ at Δ*T* of 50 K) [65], and carbon fiber (0.0101 mW m^−2^ K^−2^ at Δ*T* of 50 K) [66].

## 4. Conclusions

The mandarin peel waste was successfully converted into carbonaceous materials for thermocell electrodes. The influence of carbonization temperature on the structural, chemical properties, and thermocell performance of CMP was explored, and CMP-800 carbonized at 800 °C generated the maximum electrical power due to the combined contributions from highly porous structure and nitrogen doping. However, no enhancement of performance by KOH activation was observed. Harvesting electrical energy from various low-grade heat sources using sustainable electrode materials would provide a promising way to address global warming and energy crisis.

## Figures and Tables

**Table 1:** Surface atomic concentrations of CMP measured by XPS.

Samples	C (at. %)	O (at. %)	N (at. %)
CMP-600	85.32	13.01	1.67
CMP-700	86.35	11.68	1.96
CMP-800	86.33	12.14	1.53
CMP-900	87.83	11.4	0.77
CMP-1000	89.85	9.4	0.75

## Data Availability

The data presented in this study are available on request from the corresponding author.

## Supplementary Materials

The following are available online at https://www.mdpi.com/article/10.3390/nano13091488/s1. Figure S1. Experimental setup for measuring the temperature coefficient of redox potential of Fe(CN)_6_^3−^/Fe(CN)_6_^4−^. Thermocouples were passivated with epoxy resin, preventing them from direct exposure to electrolyte. Figure S2. Dependence of ionic conductivity of 0.4 M Fe(CN)_6_^3−^/Fe(CN)_6_^4−^ electrolyte on temperature. Figure S3. FESEM images of as-synthesized CMP-800 before washing with HCl and DI water. Figure S4. (a) FESEM and (b–d) EDS mapping images of AMP-800-1. Figure S5. XPS survey spectra of as-synthesized CMP-800 and washed CMP-800. Figure S6. Optical images of a CMP-800 electrode (a) before and (b) after ten assembly and disassembly tests, (c) power density, and (d) internal resistance of thermocell with CMP-800 electrodes according to the number of tests. Figure S7. Equivalent circuit model of a thermocell for the analysis of EIS. R_s_ denotes the ohmic resistance, R_ct_ is the charge transfer resistance, Z_w_ accounts for the Warburg impedance, and CPE is the capacitance. Figure S8. Nyquist plots of CMP-800 according to Δ*T*.

## References

[1] Chen L., Msigwa G., Yang M., Osman A.I., Fawzy S., Rooney D.W., Yap P.S. (2022). Strategies to achieve a carbon neutral society: A review. Environ. Chem. Lett..

[2] Stančin H., Mikulčić H., Wang X., Duić N. (2020). A review on alternative fuels in future energy system. Renew. Sustain. Energy Rev..

[3] Liu Y., Wang H., Sherrell P.C., Liu L., Wang Y., Chen J. (2021). Potentially Wearable Thermo-Electrochemical Cells for Body Heat Harvesting: From Mechanism, Materials, Strategies to Applications. Adv. Sci..

[4] Gunathilaka I.E., Pringle J.M., O’Dell L.A. (2021). Operando magnetic resonance imaging for mapping of temperature and redox species in thermo-electrochemical cells. Nat. Commun..

[5] Guo B., Hoshino Y., Gao F., Hayashi K., Miura Y., Kimizuka N., Yamada T. (2020). Thermocells Driven by Phase Transition of Hydrogel Nanoparticles. J. Am. Chem. Soc..

[6] Buckingham M.A., Laws K., Sengel J.T., Aldous L. (2020). Using iron sulphate to form both n-type and p-type pseudo-thermoelectrics: Non-hazardous and ‘second life’ thermogalvanic cells. Green Chem..

[7] Ikeda Y., Fukui K., Murakami Y. (2019). Integration of thermo-electrochemical conversion into forced convection cooling. Phys. Chem. Chem. Phys..

[8] Xiang Y., Guo X., Zhu H., Zhang Q., Zhu S. (2023). Aqueous biphase-boosted liquid-state thermocell for continuous low-grade heat harvesting. Chem. Eng. J..

[9] Liu Y., Zhang Q., Odunmbaku G.O., He Y., Zheng Y., Chen S., Zhou Y., Li J., Li M., Sun K. (2022). Solvent effect on the Seebeck coefficient of Fe^2+^/Fe^3+^ hydrogel thermogalvanic cells. J. Mater. Chem. A.

[10] Zhou H., Yamada T., Kimizuka N. (2016). Supramolecular Thermo-Electrochemical Cells: Enhanced Thermoelectric Performance by Host-Guest Complexation and Salt-Induced Crystallization. J. Am. Chem. Soc..

[11] He X., Sun H., Li Z., Chen X., Wang Z., Niu Y., Jiang J., Wang C. (2022). Redox-induced thermocells for low-grade heat harvesting: Mechanism, progress, and their applications. J. Mater. Chem. A.

[12] Hu R., Cola B.A., Haram N., Barisci J.N., Lee S., Stoughton S., Wallace G., Too C., Thomas M., Gestos A. (2010). Harvesting waste thermal energy using a carbon-nanotube-based thermo-electrochemical cell. Nano Lett..

[13] Yu B., Duan J., Cong H., Xie W., Liu R., Zhuang X., Wang H., Qi B., Xu M., Wang Z.L. (2020). Thermosensitive crystallization-boosted liquid thermocells for low-grade heat harvesting. Science.

[14] Kim J.H., Lee J.H., Palem R.R., Suh M.-S., Lee H.H., Kang T.J. (2019). Iron (II/III) perchlorate electrolytes for electrochemically harvesting low-grade thermal energy. Sci. Rep..

[15] Parida K., Bark H., Lee P.S. (2021). Emerging Thermal Technology Enabled Augmented Reality. Adv. Funct. Mater..

[16] Firth A., Zhang B., Yang A. (2019). Quantification of global waste heat and its environmental effects. Appl. Energy.

[17] Romano M.S., Li N., Antiohos D., Razal J.M., Nattestad A., Beirne S., Fang S., Chen Y., Jalili R., Wallace G.G. (2013). Carbon Nanotube—Reduced Graphene Oxide Composites for Thermal Energy Harvesting Applications. Adv. Mater..

[18] Kang T.J., Fang S., Kozlov M.E., Haines C.S., Li N., Kim Y.H., Chen Y., Baughman R.H. (2012). Electrical Power from Nanotube and Graphene Electrochemical Thermal Energy Harvesters. Adv. Funct. Mater..

[19] Salazar P.F., Kumar S., Cola B.A. (2012). Nitrogen- and Boron-Doped Carbon Nanotube Electrodes in a Thermo-Electrochemical Cell. J. Electrochem. Soc..

[20] Tsierkezos N.G., Knauer A., Ritter U. (2014). Thermodynamic investigation of ferrocyanide/ferricyanide redox system on nitrogen-doped multi-walled carbon nanotubes decorated with gold nanoparticles. Thermochim. Acta.

[21] Qian W., Cao M., Xie F., Dong C. (2016). Thermo-Electrochemical Cells Based on Carbon Nanotube Electrodes by Electrophoretic Deposition. Nano-Micro Lett..

[22] Zhang L., Kim T., Li N., Kang T.J., Chen J., Pringle J.M., Zhang M., Kazim A.H., Fang S., Haines C. (2017). High Power Density Electrochemical Thermocells for Inexpensively Harvesting Low-Grade Thermal Energy. Adv. Mater..

[23] Jung S.-M., Kwon J., Lee J., Shim K., Park D., Kim T., Kim Y.H., Hwang S.J., Kim Y.-T. (2020). Cu-Based Thermoelectrochemical Cells for Direct Conversion of Low-Grade Waste Heat into Electricity. ACS Appl. Energy Mater..

[24] Jung S.-M., Kwon J., Lee J., Han I.K., Kim K.-S., Kim Y.S., Kim Y.-T. (2021). Cost-efficient nickel-based thermo-electrochemical cells for utilizing low-grade thermal energy. J. Power Sources..

[25] Sonter L.J., Dade M.C., Watson J.E.M., Valenta R.K. (2020). Renewable energy production will exacerbate mining threats to biodiversity. Nat. Commun..

[26] Jia H., Wang S., Sun J., Yin K., Xie X., Sun L. (2019). Nitrogen-doped microporous carbon derived from a biomass waste-metasequoia cone for electrochemical capacitors. J. Alloys Compd..

[27] Subramani K., Sudhan N., Karnan M., Sathish M. (2017). Orange Peel Derived Activated Carbon for Fabrication of High-Energy and High-Rate Supercapacitors. ChemistrySelect.

[28] Yang V., Arumugam Senthil R., Pan J., Rajesh Kumar T., Sun Y., Liu X. (2020). Hierarchical porous carbon derived from jujube fruits as sustainable and ultrahigh capacitance material for advanced supercapacitors. J. Colloid. Interface Sci..

[29] Ma Q., Xi H., Cui F., Zhang J., Chen P., Cui T. (2022). Self-templating synthesis of hierarchical porous carbon with multi-heteroatom co-doping from tea waste for high-performance supercapacitor. J. Energy Storage.

[30] Zou K., Guan Z., Deng Y., Chen G. (2020). Nitrogen-rich porous carbon in ultra-high yield derived from activation of biomass waste by a novel eutectic salt for high performance Li-ion capacitors. Carbon.

[31] Guo D., Li Z., Liu P., Sun M. (2021). N, P, S co-doped biomass-derived hierarchical porous carbon through simple phosphoric acid-assisted activation for high-performance electrochemical energy storage. Int. J. Hydrogen Energy.

[32] Wu M.-F., Hsiao C.-H., Lee C.-Y., Tai N.-H. (2020). Flexible Supercapacitors Prepared Using the Peanut-Shell-Based Carbon. ACS Omega..

[33] Karaman C., Karaman O., Atar N., Yola M.L. (2021). Sustainable electrode material for high-energy supercapacitor: Biomass-derived graphene-like porous carbon with three-dimensional hierarchically ordered ion highways. Phys. Chem. Chem. Phys..

[34] Wang S., Zou K., Qian Y., Deng Y., Zhang L., Chen G. (2019). Insight to the synergistic effect of N-doping level and pore structure on improving the electrochemical performance of sulfur/N-doped porous carbon cathode for Li-S batteries. Carbon.

[35] Chen X., Du G., Zhang M., Kalam A., Su Q., Ding S., Xu B. (2019). Nitrogen-doped hierarchical porous carbon derived from low-cost biomass pomegranate residues for high performance lithium-sulfur batteries. J. Electroanal. Chem..

[36] Xiang J., Lv W., Mu C., Zhao J., Wang B. (2017). Activated hard carbon from orange peel for lithium/sodium ion battery anode with long cycle life. J. Alloys Compd..

[37] Nazhipkyzy M., Maltay A.B., Askaruly K., Assylkhanova D.D., Seitkazinova A.R., Mansurov Z.A. (2022). Biomass-Derived Porous Carbon Materials for Li-Ion Battery. Nanomaterials.

[38] Aruchamy K., Dharmalingam K., Lee C.W., Mondal D., Sanna Kotrappanavar N. (2022). Creating ultrahigh surface area functional carbon from biomass for high performance supercapacitor and facile removal of emerging pollutants. Chem. Eng. J..

[39] Chen Y., Zhang X., Chen W., Yang H., Chen H. (2017). The structure evolution of biochar from biomass pyrolysis and its correlation with gas pollutant adsorption performance. Bioresour. Technol..

[40] Oh W.-D., Lisak G., Webster R.D., Liang Y.-N., Veksha A., Giannis A., Moo J.G.S., Lim J.-W., Lim T.-T. (2018). Insights into the thermolytic transformation of lignocellulosic biomass waste to redox-active carbocatalyst: Durability of surface active sites. Appl. Catal..

[41] Jang S.-K., Jung C.-D., Seong H., Myung S., Kim H. (2022). An integrated biorefinery process for mandarin peel waste elimination. J. Clean. Prod..

[42] El-Kady M.F., Strong V., Dubin S., Kaner R.B. (2012). Laser Scribing of High-Performance and Flexible Graphene-Based Electrochemical Capacitors. Science.

[43] Kim J.H., Choi Y., Shin G., Jeon J.G., Kim H.J., Han Y., So B.J., Yun S., Kim T., Kang T.J. (2023). Energy harvesting from liquid cooling systems using thermo-electrochemical flow cells. J. Power Sources.

[44] Hu Y., Xie K., Wang H., Yuan C., Cao B., Qian L., Wang S., Fazeli Zafar F., Ding K., Wang Q. (2021). Preparation and property of N-doped porous carbon material by one-step pyrolysis of protein-rich algal biomass. J. Anal. Appl. Pyrolysis..

[45] Yoon S.-H., Lim S., Song Y., Ota Y., Qiao W., Tanaka A., Mochida I. (2004). KOH activation of carbon nanofibers. Carbon.

[46] Ferrari A.C. (2007). Raman spectroscopy of graphene and graphite: Disorder, electron-phonon coupling, doping and nonadiabatic effects. Solid State Commun..

[47] Deldicque D., Rouzaud J.-N., Velde B. (2016). A Raman—HRTEM study of the carbonization of wood: A new Raman-based paleothermometer dedicated to archaeometry. Carbon.

[48] Ammar M.R., Galy N., Rouzaud J.N., Toulhoat N., Vaudey C.E., Simon P., Moncoffre N. (2015). Characterizing various types of defects in nuclear graphite using Raman scattering: Heat treatment, ion irradiation and polishing. Carbon.

[49] Wang C., Wang H., Dang B., Wang Z., Shen X., Li C., Sun Q. (2020). Ultrahigh yield of nitrogen doped porous carbon from biomass waste for supercapacitor. Renew. Energy.

[50] Zhang Y., Liu X., Wang S., Dou S., Li L. (2016). Interconnected honeycomb-like porous carbon derived from plane tree fluff for high performance supercapacitors. J. Mater. Chem. A.

[51] Liu M., Zhang Z., Dou M., Li Z., Wang F. (2019). Nitrogen and oxygen co-doped porous carbon nanosheets as high-rate and long-lifetime anode materials for high-performance Li-ion capacitors. Carbon.

[52] Hou L., Hu Z., Wang X., Qiang L., Zhou Y., Lv L., Li S. (2019). Hierarchically porous and heteroatom self-doped graphitic biomass carbon for supercapacitors. J. Colloid. Interface Sci..

[53] Yang S., Wang S., Liu X., Li L. (2019). Biomass derived interconnected hierarchical micro-meso-macro-porous carbon with ultrahigh capacitance for supercapacitors. Carbon.

[54] Zhao F., Chen S., Xiang H., Gao T., Wang D., Wei D., Sillanpää M., Ke Y., Tang C.-J. (2022). Selectively capacitive recovery of rare earth elements from aqueous solution onto Lewis base sites of pyrrolic-N doped activated carbon electrodes. Carbon.

[55] Liu D., Zhang X., Sun Z., You T. (2013). Free-standing nitrogen-doped carbon nanofiber films as highly efficient electrocatalysts for oxygen reduction. Nanoscale.

[56] Zhou N., Wang N., Wu Z., Li L. (2018). Probing Active Sites on Metal-Free, Nitrogen-Doped Carbons for Oxygen Electroreduction: A Review. Catalysts.

[57] Xu G.H., Chen J.C., Liu D.H., Zhang Y.H., Jiang P., Ye X.Q. (2008). Minerals, phenolic compounds, and antioxidant capacity of citrus peel extract by hot water. J. Food Sci..

[58] Lu X.P., Li F.F., Xiong J., Cao X.J., Ma X.C., Zhang Z.M., Cao S.Y., Xie S.X. (2017). Transcriptome and Metabolome Analyses Provide Insights into the Occurrence of Peel Roughing Disorder on Satsuma Mandarin (*Citrus unshiu* Marc.) Fruit. Front Plant Sci..

[59] Zhu P., Zhao Y. (2019). Cyclic voltammetry measurements of electroactive surface area of porous nickel: Peak current and peak charge methods and diffusion layer effect. Mater. Chem. Phys..

[60] Duan J., Yu B., Huang L., Hu B., Xu M., Feng G., Zhou J. (2021). Liquid-state thermocells: Opportunities and challenges for low-grade heat harvesting. Joule.

[61] Zhou M., Pu F., Wang Z., Guan S. (2014). Nitrogen-doped porous carbons through KOH activation with superior performance in supercapacitors. Carbon.

[62] Xu B., Wu F., Su Y., Cao G., Chen S., Zhou Z., Yang Y. (2008). Competitive effect of KOH activation on the electrochemical performances of carbon nanotubes for EDLC: Balance between porosity and conductivity. Electrochim. Acta.

[63] Wei S., Ma J., Wu D., Chen B., Du C., Liang L., Huang Y., Li Z., Rao F., Chen G. (2023). Constructing Flexible Film Electrode with Porous Layered Structure by MXene/SWCNTs/PANI Ternary Composite for Efficient Low-Grade Thermal Energy Harvest. Adv. Funct. Mater..

[64] Zhou Y., Qian W., Huang W., Liu B., Lin H., Dong C. (2019). Carbon Nanotube-Graphene Hybrid Electrodes with Enhanced Thermo-Electrochemical Cell Properties. Nanomaterials.

[65] Dong M., Qian W., Liu X., Chen Y., Huang W., Dong C. (2022). Enhanced thermo cell properties from N-doped carbon nanotube-Pd composite electrode. Surf. Coat. Technol..

[66] Artyukhov D., Kiselev N., Gorshkov N., Kovyneva N., Ganzha O., Vikulova M., Gorokhovsky A., Offor P., Boychenko E., Burmistrov I. (2021). Harvesting Waste Thermal Energy Using a Surface-Modified Carbon Fiber-Based Thermo-Electrochemical Cell. Sustainability.

