# Supplementary material for: Biomass-Derived Sustainable Electrode Material for Low-Grade Heat Harvesting
Source: Nanomaterials (Basel). 2023 Apr 27;13(9):1488. doi: 10.3390/nano13091488 (PMC10180141; doi:10.3390/nano13091488)
Supplement: Supplementary file 1 [file nanomaterials-13-01488-s001.zip › nanomaterials-2343573-supplementary.pdf]

Supplementary Materials

Biomass-derived sustainable electrode material for low-grade heat harvesting

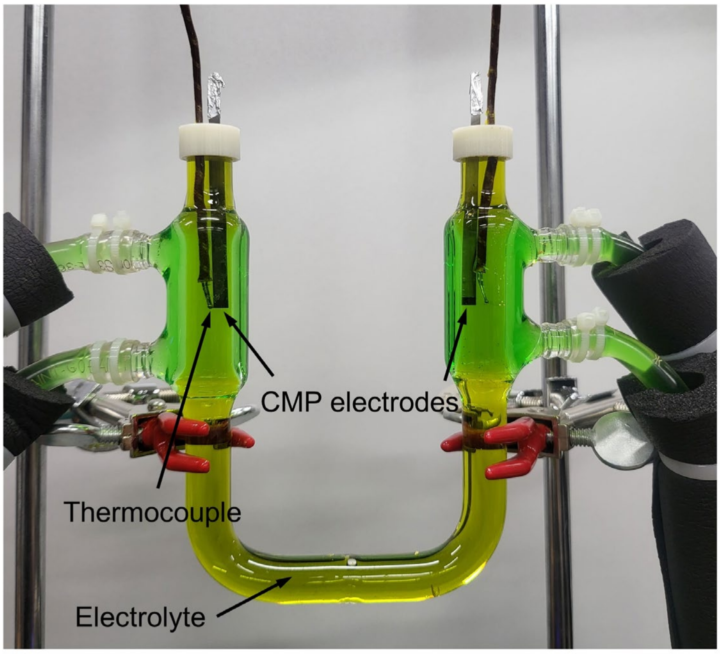

Figure S1. Experimental setup for measuring the temperature coefficient of the redox potential of $Fe(CN)_6^{3-}$/ $Fe(CN)_6^{4-}$. Thermocouples were passivated with epoxy resin, preventing them from directly exposing the electrolyte.

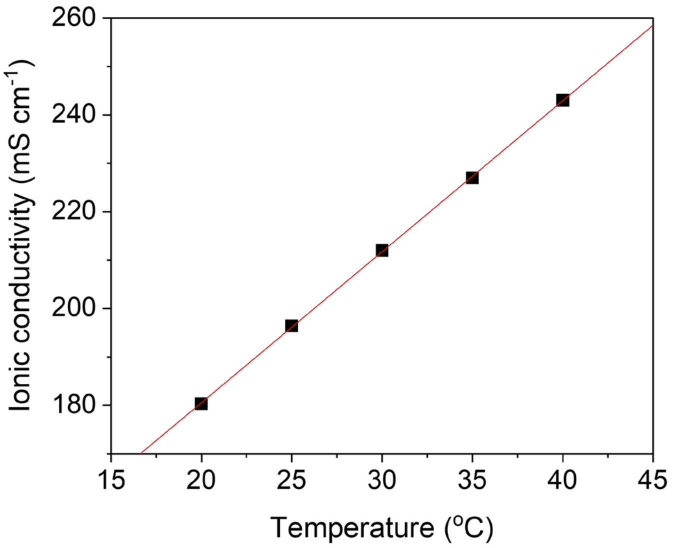

Figure S2. Dependence of ionic conductivity of 0.4 M $Fe(CN)_6^{3-}/Fe(CN)_6^{4-}$ electrolyte on temperature.

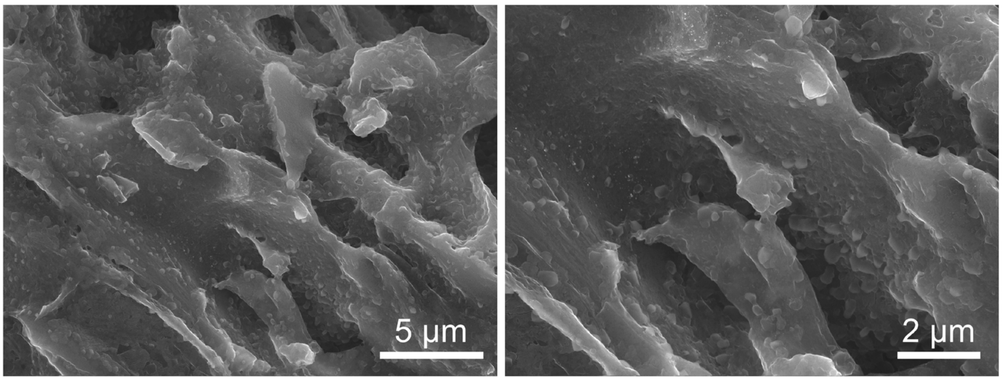

Figure S3. FESEM images of as-synthesized CMP-800 before washing with HCl and DI water.

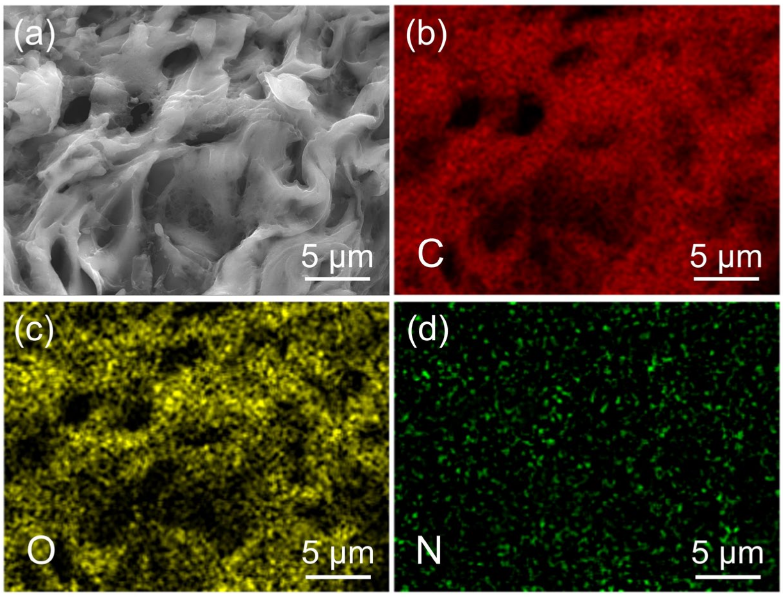

Figure S4. (a) FESEM and (b-d) EDS mapping images of AMP-800-1.

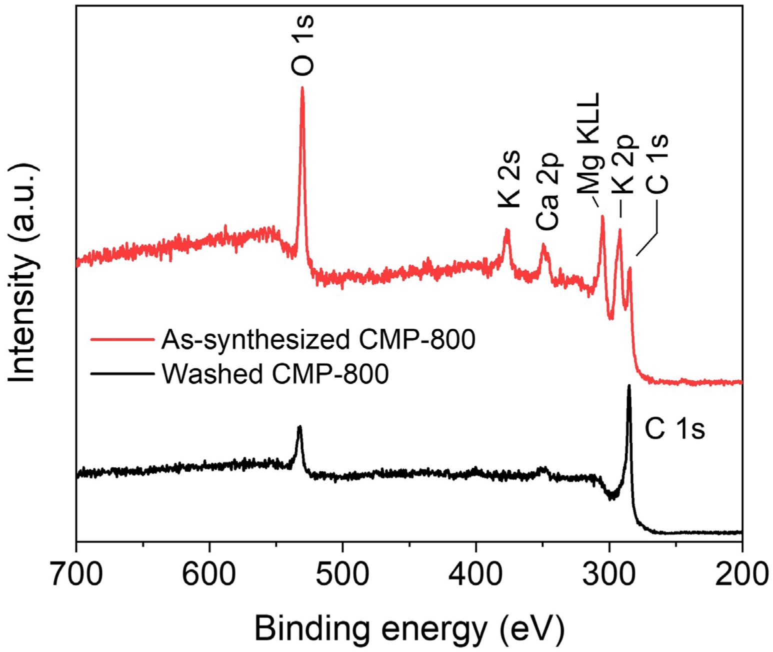

Figure S5. XPS survey spectra of as-synthesized CMP-800 and washed CMP-800.

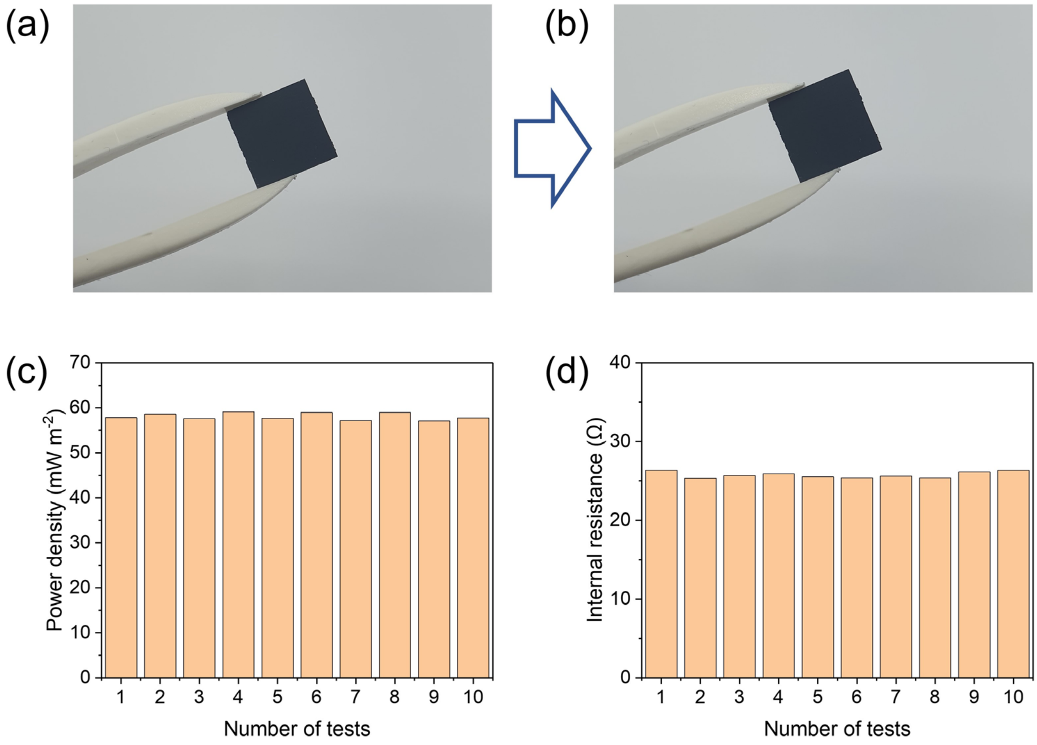

Figure S6. Optical images of a CMP-800 electrode (a) before and (b) after ten assembly and disassembly tests, (c) power density, and (d) internal resistance of thermocell with CMP-800 electrodes according to the number of tests.

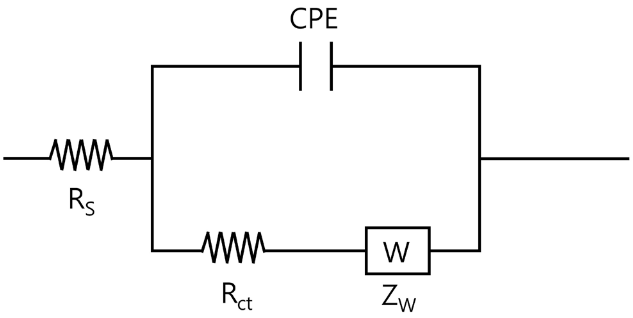

Figure S7. Equivalent circuit model of a thermocell for the analysis of EIS. R_s denotes the ohmic resistance, R_{ct} is the charge transfer resistance, Z_w accounts for the Warburg impedance, and CPE is the capacitance.

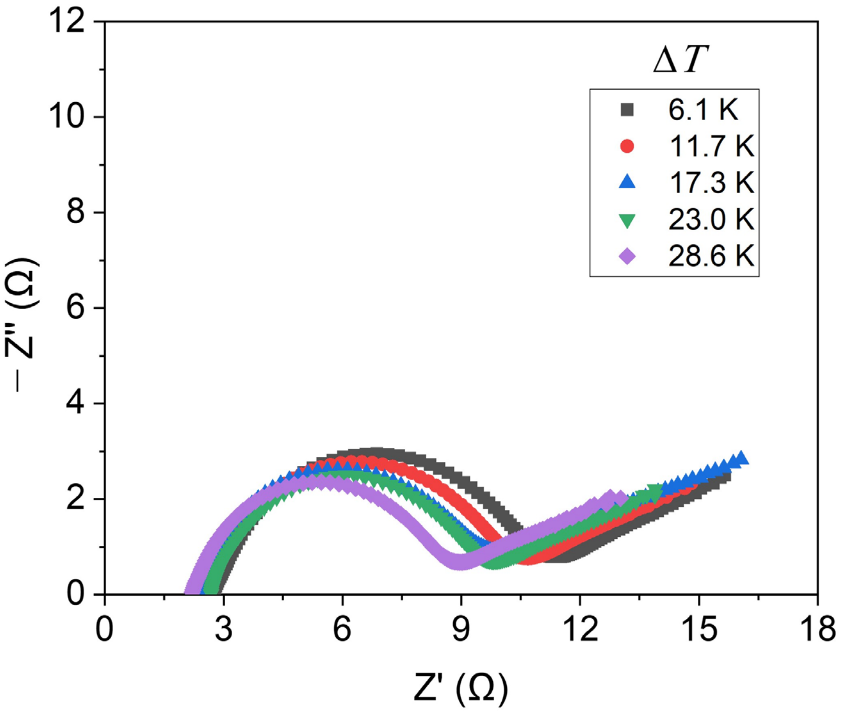

Figure S8. Nyquist plots of CMP-800 according to Δ*T*.
